# Neuropuncture, an Effective Treatment Method for Patients with Subjective Tinnitus Accompanied with Hearing Loss: Case Reports

**DOI:** 10.1089/acu.2020.1514

**Published:** 2021-08-17

**Authors:** Helen K. Law, Michael D. Corradino

**Affiliations:** ^1^ Chinese Acupuncture Healthcare, LLC, Princeton, NJ, USA.; ^2^ Neuropuncture, Inc., Boynton Beach, FL, USA.

**Keywords:** tinnitus, hearing loss, SNHL, acupuncture, electroacupuncture, Neuropuncture

## Abstract

***Background:*** Tinnitus is a serious health condition. It can be debilitating and as such negatively affects a patient's quality of life. People with tinnitus often experience distress, depression, anxiety, frequent mood swings, sleep disturbances, irritability, frustration, poor concentration, and possible suicidal thoughts or actions.

***Objective:*** The goal of this article is to introduce an acupuncture system, based on neurophysiology and termed Neuropuncture, as a possible effective treatment method for tinnitus accompanied with and/or secondary to hearing loss. The treatment protocol works by targeting the greater auricular nerve, trigeminal nerve, cervical plexus, and auditory cortex to neuromodulate, neurorehabilitate, and neuroregulate the nervous system and repair the nerve damage.

***Design:*** Three case studies are presented herein as examples. They are based on neurophysiologic mechanism of tinnitus and hearing loss, treatment principle, treatment methods, and subjective and clinically objective tests. Electroacupuncture protocols used various frequencies with microcurrent and millicurrent stimulation.

***Conclusions:*** Neuropuncture system is an effective treatment for patients with acute and chronic subjective tinnitus and hearing loss. Results showed reduction of tinnitus and partial restoration of hearing loss. Further research and possible large-scale trial studies are suggested.

## Introduction

Tinnitus is the perception of sound when there is an absence of actual external noise.^1^ Approximately 15% of American adults report some trouble of hearing and roughly 10% of the U.S. population, or about 25 million people, struggle with burdensome chronic tinnitus, whereas 2 million suffer from extreme and debilitating cases. It is estimated 90% of tinnitus cases occur with underlying hearing loss.^2^

Tinnitus is commonly described as ringing in the ears. It can also sound like roaring, hissing, buzzing, whistling, swooshing, ringing, or clicking. It may be soft or loud, and high or low pitched, and can happen in 1 or both ears. It can be the result of a number of health conditions but is not a disease by itself.^3^ Tinnitus is considered as a pathology that involves synaptic plasticity at the level of the synapses between inner hair cells and the auditory nerve.^4^ Specifically when sound waves hit the tympanic membrane, the vibration causes the Organ of Corti and its hair cells to generate electrical signals that are then sent to the brain for interpretation. The tympanic membrane is innervated by the auriculotemporal branch of the trigeminal nerve, the auricular branch of the facial nerve, the auricular branch of the vagus nerve, and the glossopharyngeal nerve.^5^

Sensory innervation to the external ear is supplied by both cranial and spinal nerves. Branches of the trigeminal, facial, and vagus nerves (CNV, VII, X) are the cranial nerve components, while the lesser occipital and greater auricular (C2, C3) nerves are the spinal nerve components involved. The vestibulocochlear nerve, also known as cranial nerve 8 (CN VIII), has distinct nuclei within the brainstem and the cochlear nerve is responsible for hearing.^6^

Recent studies have shown that acupuncture could regulate inflammatory response,^7^ and enhance the excitability and conductivity of the auditory nerve.^8^ Karavis reported how the neurophysiology mechanism of acupuncture activates neural pathways that created multifactorial phenomenon throughout the body.^9^ Han concluded that acupuncture or electrical stimulation in specific frequencies applied to certain body sites can facilitate the release of specific neuropeptides in the central nervous system (CNS), which subsequently elicit physiologic effects and activate self-healing mechanisms.^10–14^ Kim et al.^15^ in a recent trial study showed that electroacupuncture is more effective than manual acupuncture and TENS (transcutaneous electrical nerve stimulation) for people with chronic tinnitus.

Based on these theories and clinical experience, the authors report herein the use of this acupuncture protocol and preliminary results on patients with acute and chronic tinnitus secondary to hearing loss treatments as a possible effective and clinically reproducible treatment method.

### Intervention

For tinnitus and hearing loss, the treatment principles are focused on local inflammation; target specific nerves such as the greater auricular nerve, facial nerve, trigeminal nerve, and vagus nerve; target specific neural plexuses that innervate the vestibulocochlear nerve, and also target the CNS specifically the auditory cortex. This acupuncture system utilizes electroacupuncture to neuromodulate the damaged nerves, neuroregulate the neural plexuses, and neurorehabilitate regions of the brain to repair them back to health.

**FIG. 1.**
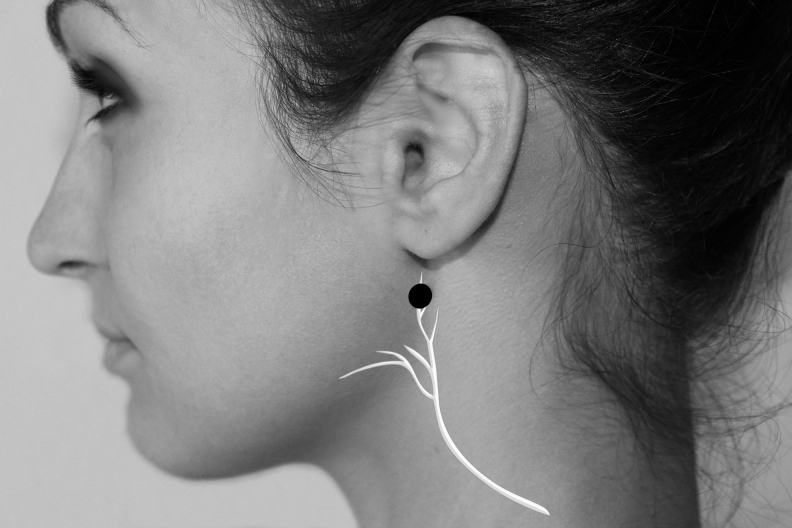
Greater auricular neuropuncture acupoint.

**FIG. 2.**
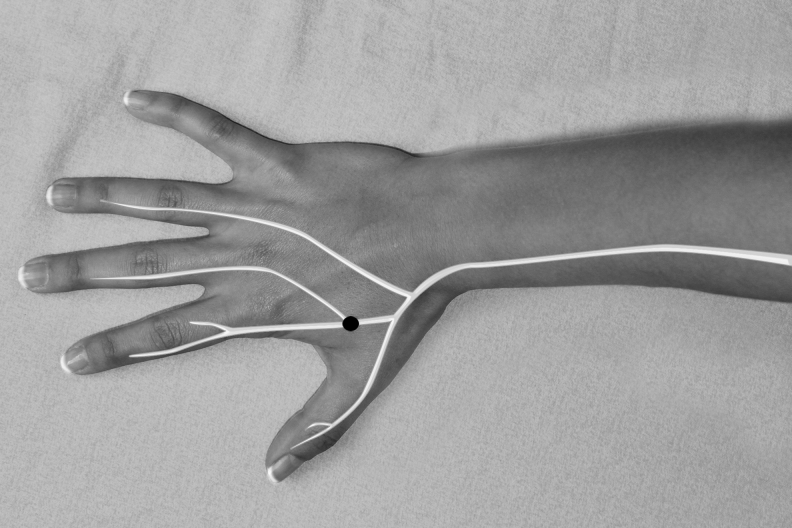
Superficial radial neuropuncture acupoint.

**FIG. 3.**
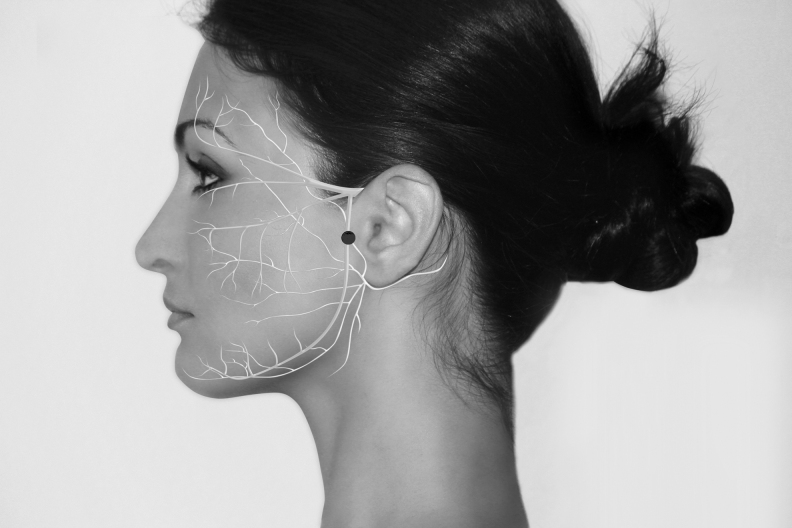
Trifacial neuropuncture acupoint.

**FIG. 4.**
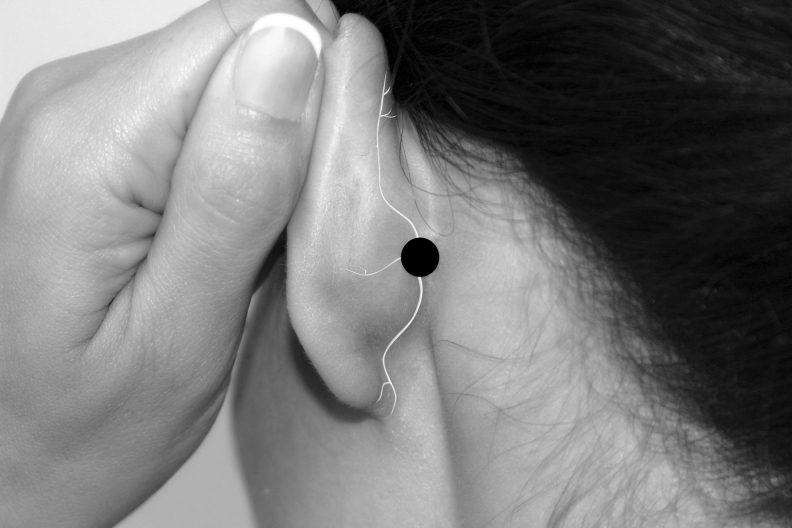
Postauricular neuropuncture acupoint.

**FIG. 5.**
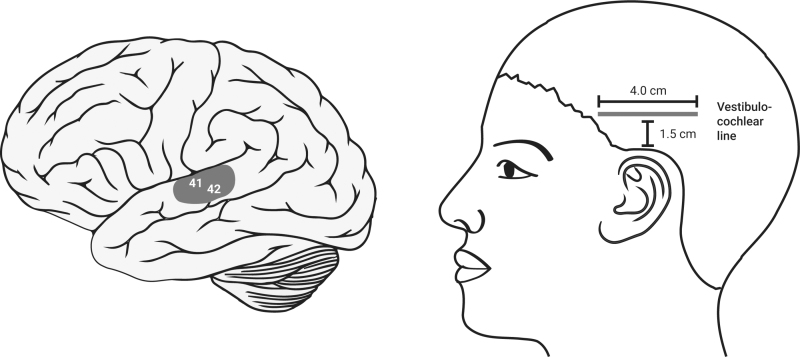
Auditory cortex-scalp acupuncture auditory and dizzy line.

The authors used 7 Neuropuncture points.^16^ These points are located near regions of the TCM (Traditional Chinese Medicine) points. They are GANP (Greater Auricular Neuropuncture Point) (Fig. 1), SRNP (Superficial Radial Neuropuncture Point) (Fig. 2), TriFNP (Trigeminal/Facial Neuropuncture Point) (Fig. 3), PANP (Posterior Auricular Neuropuncture Point) (Fig. 4) of the affected side, HTJJ (Hua Tuo Jia Ji acupuncture points) C2/C3, and the Neuropuncture Scalp Auditory Cortex (Neuropuncture Scalp Auditory Cortex) (Fig. 5) of both sides, using sterile single-use 0.25-mm-thick 50-mm-long needles (DBC Spring Ten Korean brand); and Pantheon electrostimulator 8c Pro. This device is manufactured by Pantheon Research in the United States and is Food and Drug Administration (FDA)-registered electrical stimulator specifically for electrical acupuncture. It provides a biphasic electrical square waveform with a 400 μs pulse width. It offers both millicurrent and microcurrent options. The frequency generator is microprocessor calibrated for most accuracy. The Neuropuncture points were selected according to neuroscience research and neuroanatomy. The depth and direction of insertion of the needle differed depending on the location of the Neuropuncture points. These acupuncture needles were applied until the patient experienced the classical De Qi sensation and then connected to electrical leads.

The main Neuropuncture prescription for tinnitus is to connect the lead with electrical clamps to GANP and SRNP, and another lead to TriFNP to PANP on the affected side. The Neuropuncture Electrical Dosage is 25 Hz microcurrent. In an alternate modified Neuropuncture prescription for tinnitus the third lead is connected from HTJJ C2 and C3 to the Neuropuncture Scalp Auditory Cortex region bilaterally. See Table 1 for details. The Neuropuncture Electrical Dosage is 2 Hz millicurrent for 30 minutes. Neuropuncture therapy was suggested for twice per week. The Subjective Units of Distress (SUD) scale used in the following case studies describes the approximate level of hearing disturbance and discomfort subjectively from the annoying noise in the ears that cause irritability, insomnia, and stress. Henry et al.^17^ concluded neither the loudness nor other psychoacoustic measures of tinnitus had a consistent relationship to the severity of this condition, hence there was no standard protocol for tinnitus evaluation.

**Table 1.** Neuropuncure Rx for Tinnitus and Hearing Loss

Disease/condition	Neuropuncture Rx	EA lead placement	Neuropuncture dosage	Commentary
Tinnitus main Rx: (1) and (2) Tinnitus modified Rx: (3) and (4)	(1) GANP-SRNP (affected ear): EA (2) TriFNP-PANP (affected ear): EA (3) GANP-TriFNP (affected ear): EA (4) HTJJ C2/C3-auditory cortex (on scalp) (B): EA	(1) Use 1 lead to connect the affected ear. (2) Use 1 lead to connect the affected ear. (3) Use 1 lead to connect the affected ear. (4) Use 2 leads to connect both sides.	(1) and (2) and (3) EA: 25 Hz microcurrent for 30 minutes. (4) EA: 2 Hz millicurrent for 30 minutes. 2 times per week for 6 weeks. Alternate main and modified Rx	This neuropuncture Rx targets the cranial nerves as well as the auditory cortex to rehabilitate and neuromodulate the affected nerves.

GANP, greater auricular neuropuncture point; PANP, Posterior Auricular Neuropuncture Point; SRNP, superficial radial neuropuncture point; TriFNP, Trigeminal/Facial Neuropuncture Point.

## Case Studies

Table 2 is a summary of the records of 14 patients, collected in the past 2 years. Three of these cases are described in detail in the following as examples for specific illustration.

**Table 2.** Summary of 14 Patients Treatment Records

	Gender	Age	Symptoms	Date of service	Treatment protocol(s)	Frequency of visits	Follow-up results
1	Male	28	Acute severe tinnitus, military personnel after shooting practice	April 2018	Tinnitus main	2 times a week for 1 week	Tinnitus completely stopped after 2 treatments. No follow-up.
2	Male	54	Tinnitus, left ear acoustic neuroma; facial droop; treated with proton beam radiation	November 2018	Tinnitus main; add treatment for facial paralysis	3 times a week for 3 weeks	Two years follow-up tinnitus was 60% reduced, facial droop 90% improved, result stayed the same since treatment
3	Male	71	Chronic tinnitus	April 2019	Tinnitus main	Once a week for 4 weeks	One year follow-up tinnitus happened only occasionally
4	Female	59	Tinnitus, severe sinusitis, loss of hearing	June 2019	Tinnitus main alternate with tinnitus modified	Once a week for 12 weeks	One year follow-up tinnitus was gone, hearing improved 90%, sinus congestion was gone
5	Female	67	Tinnitus, chronic allergic rhinitis and ear congestion	June 2019	Tinnitus main	Once a week for 12 weeks, then once a week for 3 months and once a month maintenance	Tinnitus was mostly not noticeable, postnasal drip continued, and ear congestion reduced
6	Female	81	Pulsatile tinnitus	November 2019	Tinnitus main	2 times a week for 3 weeks	One year follow-up tinnitus was 90% improved
7	Female	58	Acute tinnitus	January 2020	Tinnitus main	2 times a week for 4 weeks	One year follow-up tinnitus was gone
8	Male	44	Tinnitus for 4 years, vertigo, neck pain, loss of balance	January 2020	Tinnitus main	Once a week for 5 weeks	One year follow-up showed tinnitus occurred occasionally, vertigo was gone, balance improved
9	Female	49	Tinnitus, C6/7 herniation, neck pain	February 2020	Tinnitus main	Once a week for 3 weeks	Tinnitus was gone after 3 weeks. One year follow-up showed the result remain the same
10	Female	47	Tinnitus, hearing loss; bartender worked in a very noisy loud place	February 2020	Tinnitus main alternate with tinnitus modified	Once a week for 5 weeks	Tinnitus and hearing loss recovered after treatment and lasted for 4 more months. However, tinnitus returned without continued treatment
11	Female	54	Tinnitus, complete loss of hearing post fourth craniectomy	July 2020	Tinnitus main alternate with tinnitus modified	Two times a week for 6 weeks, then once a week for maintenance	6 months follow-up, tinnitus is gone, hearing continued improving
12	Male	56	Tinnitus, fullness in the right ear, hole in right ear drum	July 2020	Tinnitus main	2 times a week for 4 weeks, then once a week for 4 weeks	6 months follow-up, fullness in the right ear completely gone, tinnitus was improved 90%
13	Male	71	Tinnitus, hearing loss, dizziness after an intense workout routine	October 2020	Tinnitus main alternate with tinnitus modified	Too times a week for 2 weeks	Tinnitus, dizziness, hearing loss completely recovered. 3 months follow-up results remained same.
14	Female	50	Tinnitus, ear congestion after fighting a flu episode	January 2021	Tinnitus main	Once a week for 4 weeks	Follow-up tinnitus reduced 80%, congestion was 90% improved

### Case 1: Tinnitus and Hearing Loss as a Result from Severe Sinus and Ear Congestion

A 57-year-old woman came to the office on June 19, 2019 seeking relief from her tinnitus and hearing loss. Her vital signs, including blood pressure and pulse rates, were within normal limits. Her current medical history included severe sinusitis, insomnia, fatigue, and stress.

One year ago, patient started to feel fullness in her left ear and experienced a high pitch ringing and vertigo. She felt very congested in her frontal sinuses and both ears, with the left ear being worse. She then developed a very loud high-pitch noise with hearing loss in her left ear at an SUD scale of 8/10. The noise interrupted her sleep at night. Patient went to her ear, nose, and throat (ENT) doctor, an audiogram was performed and showed significant hearing loss in her left ear, registering 40 dB within the range of 250 to 4,000 Hz, with rapid reduction beyond it.

A main Neuropuncture tinnitus prescription was performed at the first treatment. Patient reported that hearing began to return slowly afterward. After 3 treatments, she reported that she was able to hear a different “layer” of sound (she is a musician) and began to enjoy listening to music again. A total 12 treatments twice a week, including Tinnitus Main and Modified Protocols, were conducted. Patient reported having tremendous “draining” from her ears and the ringing changed from high pitch to a different lower tone with much less intensity with an SUD scale of 4/10. Her relationship with her husband improved because she did not have to constantly ask him to repeat himself in conversation.

The patient saw her ENT doctor for her 1 year follow-up in June, 2020. The result of the audiology test showed a significant improvement in hearing of her left ear. She was able to hear low frequency at 250 Hz clearly and high frequencies ranging from 4,000 to 6,000 Hz at 15 dB. Eighteen months follow-up in the office showed the tinnitus was occasionally noticeable; her SUD scale was at 1/10 and her hearing continued to improve.

### Case 2: Tinnitus and Hearing Loss Postcraniectomy

A 54-year-old woman came to the office on July 21, 2020 reporting that she had completely lost hearing in the left ear. Her vital signs, including blood pressure and pulse rate, were within normal limits. Her current medical history includes loss of hearing in the left ear, tinnitus with low-pitch roaring noise at an SUD scale of 6/10, insomnia, headaches, blurry vision with nystagmus, severe sinus congestion, neck pain, and hot flashes.

The loss of hearing happened immediately after her fourth craniectomy for resection of posterior fossa recurrent ependymoma located in the left lateral recess of the fourth ventricle. She was diagnosed with unilateral sensorineural hearing loss (SNHL).

Before the first Neuropuncture treatment, a quick rudimentary test was conducted to check the ability of hearing by clicking the fingernails outside the left ear. Patient reported not being able to hear anything. Neuropuncture tinnitus main protocol was used at the first session. After completing the treatment, again using the same finger clicking test, the patient reported that she was able to regain some hearing. The sinus congestion was much reduced after the second treatment. After the third treatment, she was able to hear conversation if spoken close to the left ear. Patient reported that the low-pitch roaring noises had significantly reduced to an SUD scale of 2/10. She also reported that she was able to hear partial conversations between family members and sometimes understood news reporting on the television.

An audiogram test was performed on August 21, 2020 after 9 treatments and that included Tinnitus Main and Modified Protocols. Patient showed improvement of her left ear that she could hear from 500 to 2,000 Hz at 20 dB. She still showed poor hearing for the low- and high-pitch sound, but this was certainly better than the complete loss of hearing in July 2020.

### Case 3: Tinnitus and Ear Congestion due to Hole in Ear Drum

A 52-year-old man came to the office on July 2, 2020 complaining of tinnitus and fullness in his right ear. His vital signs, including blood pressure and pulse rate, were within normal limits. Patient's current medical history includes right ear tinnitus, loss of hearing, with a fullness sensation.

He reported feeling fluid in his right ear back in 2004, and his ENT doctor implanted an ear tube to help drain the fluid. This ear tube was removed in 2012. Patient started having tinnitus in 2016. He visited his ENT doctor who found a hole in the ear drum in 2018. Patient continued feeling the fullness in his right ear with high pitch ringing sound at an SUD scale of 6/10. In June 2020, the ENT doctor suggested inserting another ear tube and prescribed steroid medicine. However, patient decided to pursue the acupuncture route.

After the first Neuropuncture tinnitus prescription treatment, patient reported feeling immediate relief, with the right ear fullness reduced, and the intensity of the high pitch dropped 2 U in the SUD scale from 6/10 to 4/10. The Neuropuncture treatment plan was 6 weeks for a total of 12 treatments that included Tinnitus Main and Modified protocols. At the end of the treatment plan on September 24, 2020 the patient reported that the tinnitus was noticeable reduced to an SUD scale of 1/10, and he only experienced slight “sloshing/flapping” sound once or twice a day.

## Discussion

Neuropuncture therapy based on neuroscience and neurophysiology target the damaged nerves by stimulating the neural plexus, spinal nerves, and the auditory cortex in the CNS to neurorehabilitate, neuromodulate, and neuroregulate the ear back to health. The 3 case reports discussed earlier show acute and chronic subjective tinnitus and/or hearing loss with very different complaints and causes. The first case patient suffered long-term severe sinusitis, the second case patient suffered hearing loss and tinnitus postcraniectomy, and the third case patient had fullness in the ear with a puncture to the eardrum. Additional cases presented in Table 2 also bring up the complexity and persistence of the causes such as the environment being a constant stimulant, for example, the bartender working in a noisy and loud workplace. The resilience in maintaining the treatment also reduced with age. All these special situations would require adherence to periodic maintenance programs.

## Conclusion

Tinnitus can negatively affect the quality of life. Hearing loss is a major public health issue. Goman et al. estimated that during the next 43 years, the number of people with hearing loss in the United States is projected to almost double.^18^ Unfortunately, there are no drugs or effective therapies to benefit those people who are suffering. Based on the 3 representative case reports discussed in detail, together with additional cases in Table 2, the main Neuropuncture tinnitus prescription and its modification are an effective treatment to help patients who suffer from acute or chronic subjective tinnitus and/or secondary to hearing loss are demonstrated. The authors are encouraged that these results offer promising potential for short- and long-terms benefits and suggest serious consideration by the medical community to conduct large-scale trial studies for further exploration.
